# A Structured Scaffold Featuring Biomimetic Heterogeneous Architecture for the Regeneration of Critical-Size Bone Defects

**DOI:** 10.3389/fbioe.2022.927050

**Published:** 2022-07-22

**Authors:** Lingjun Wang, Jiannan Mao, Feng Cai, Jincheng Tang, Kun Xi, Yu Feng, Yichang Xu, Xiao Liang, Yong Gu, Liang Chen

**Affiliations:** ^1^ Department of Orthopaedics, The First Affiliated Hospital of Soochow University, Suzhou, China; ^2^ Department of Orthopaedics, The Affiliated Jiangyin Hospital of Nantong University Medical College, Jiang Yin, China

**Keywords:** structured scaffold, osteogenesis, angiogenesis, bone defect, biomaterial

## Abstract

The regeneration of critical-size bone defects on long bones has remained a significant challenge because of the complex anatomical structure and vascular network. In such circumstances, current biomaterial forms with homogeneous structure and function can hardly satisfy the need for both osteogenesis and angiogenesis. In the current study, a heterogeneous biomimetic structured scaffold was constructed with the help of a 3D printed mold to simultaneously mimic the outer/inner periosteum and intermediate bone matrix of a natural long bone. Because of the reinforcement *via* modified mesoporous bioactive glass nanoparticles (MBGNs), enhanced structural stability and adequate osteogenic capacity could be achieved for the intermediate layer of this scaffold. Conversely, GelMA incorporated with VEGF-loaded liposome exhibiting controlled release of the angiogenic factor was applied to the inner and outer layers of the scaffold. The resulting heterogeneous structured scaffold was shown to successfully guide bone regeneration and restoration of the natural bone anatomic structure, rendering it a promising candidate for future orthopedic clinical studies.

## Introduction

As the most common trauma condition, bone fractures have garnered increasing attention over time. Although most fractures heal adequately because of the self-healing capacity of the human body, around 5–10% of fractures suffer from delayed union and nonunion, especially in the presence of diseases such as osteoporosis and diabetes (Zura et al., 2016). The inability to achieve effective fracture healing usually requires multiple surgeries and prolonged hospitalization, which would bring a dramatic burden on patients. Therefore, the treatment of bone defects has been a key area of investigation in orthopedic clinics. From the perspective of anatomy, bone tissue is characterized by its heterogeneous structure composed of outer periosteum, cortical bone, and inner cancellous bone covered by the inner periosteum (Clarke, 2008). Such structures are also responsible for the complex blood supply system in bone tissue which is frequently damaged in trauma conditions (Marenzana and Arnett, 2013). However, current regenerative biomaterials usually focus on reconstructing bone tissue while overlooking the restoration of the normal anatomic structure and blood supply system of a fractured bone (Habibovic and Barralet, 2011; Matassi et al., 2011).

The importance of the periosteum in fracture healing has been well recognized (Aro et al., 1990; Neagu et al., 2016). The angiogenic activity guided by the periosteum plays an essential role in the revascularization and reconstruction of damaged bone tissue (Nobuto et al., 2005; Zhang et al., 2008). Numerous attempts have been reported to reconstruct the damaged periosteum at the fracture site using biomedical materials (Li et al., 2016). Electrospun fiber membranes have been reported to act as an artificial periosteum in bone tissue engineering, producing satisfying outcomes in preliminary studies (Gong et al., 2018; Liu et al., 2020). However, the basic form of artificial membrane alone is not adequate for treating critical-size bone defects because of its incapacity to fill defect areas. Combining such membrane-shaped biomaterials with additional bulk materials could be the solution (Zurina et al., 2020). High complexity resulting from the introduction of two distinct forms of materials could still be a persistent problem for its adoption in clinical practice. Designing one scaffold capable of simultaneously acting as an osteogenic matrix and an angiogenic periosteum could represent a potentially ideal solution for this problem.

As a modification of gelatin *via* methacrylic anhydride functionalization, GelMA (gelatin methacryloyl) has been recognized as an excellent platform to mimic the natural extracellular matrix because of the abundance of the RGD (arginine-glycine-aspartate) domain on its molecular chain (Yue et al., 2015). Its potential as a template for osteogenesis and angiogenesis has made GelMA a major topic of interest in tissue engineering (Stratesteffen et al., 2017; Anada et al., 2019). UV light triggers GelMA crosslinking and allows for convenient application in clinical practice. However, the innate nature of the hydrogel material has rendered GelMA with poor mechanical properties, which is unsuitable for the engineering of tough tissue such as bone. Furthermore, because of the loose and water-rich structure of hydrogels, GelMA (as a drug-loading platform) cannot maintain the sustained release of bioactive factors to match the lengthy process of bone regeneration (Lai et al., 2016). Further modification and functionalization would be required to fit the GelMA hydrogel for the mission.

Nanomaterials have garnered increased attention among the various strategies attempting to enhance soft hydrogel’s mechanical and biological function because of their high effectiveness and diverse functionalities (Kurian et al., 2022; Sakr et al., 2022). Previously, our group enhanced hydrogels with various nanomaterials. By incorporating hydrogels with surface-modified mesoporous bioactive glass, we have endowed GelMA hydrogel with enhanced mechanical strength and osteogenic potential (Xin et al., 2017). In addition, liposomes were also employed in the modification of GelMA hydrogels and they were found to enhance the structural integrity of GelMA and allow for the loading of multiple drugs for controlled release (Cheng et al., 2018). Because of the strategies mentioned earlier, GelMA enhanced using diverse nanomaterials was adequate to serve as an ECM template and drug reservoir in bone tissue engineering (Gong et al., 2019; Shao et al., 2019). However, mimicking the natural anatomic structure of bone requires much more than tunable mechanical strength and controlled release ability. Recreation of the natural anatomic structure has increased the demand for GelMA hydrogel, which is usually used as a homogeneous bulk material (Klotz et al., 2016). Briefly, GelMA has suffered from its incapacity to construct sophisticated biomimetic structures. This is because the homogeneous architecture of the GelMA scaffolds fails to mimic the heterogeneous structure of natural bone, thus hindering its potential to treat critical-size bone defects (Zhang et al., 2021).

**FIGURE 1:**
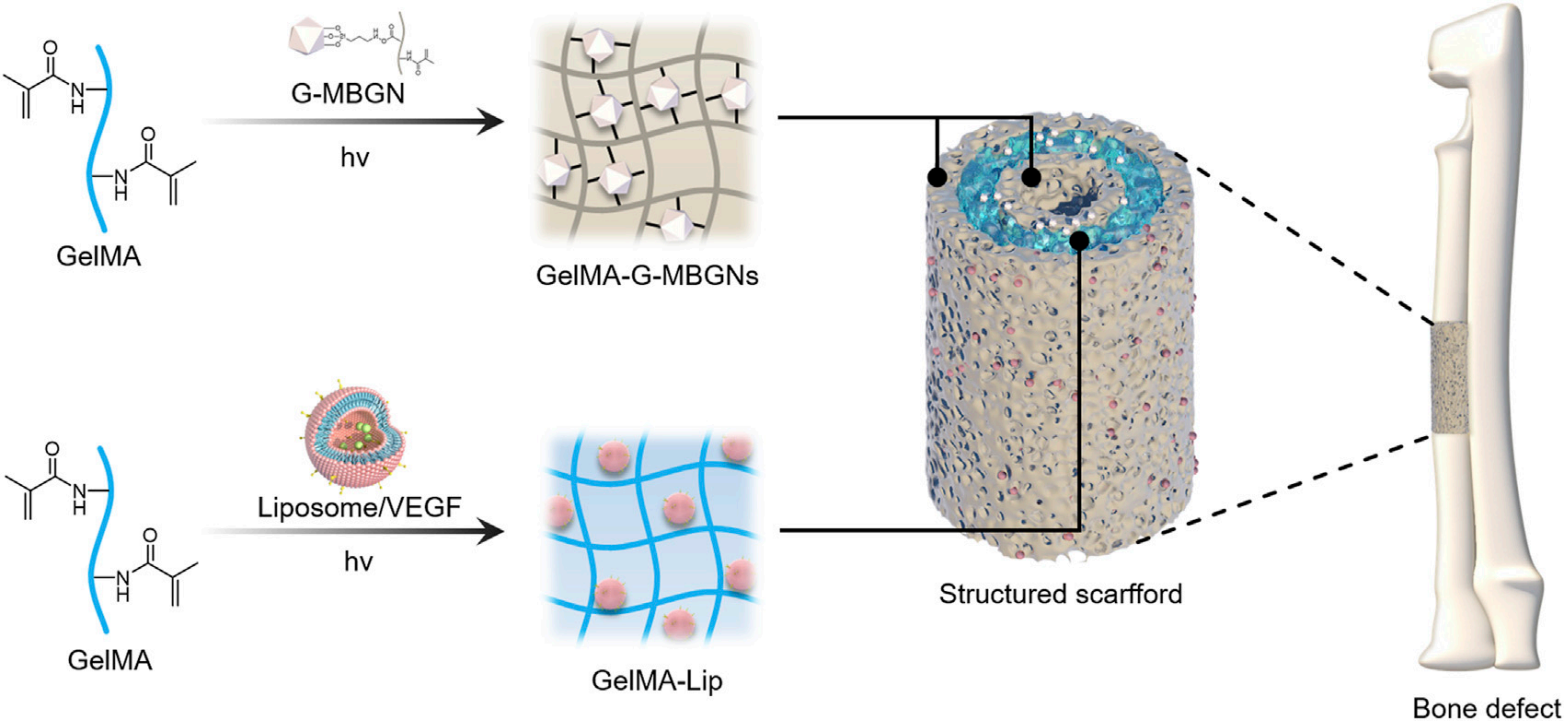
Schematic illustration.

In this study, to address the drawbacks associated with traditional bulk biomaterials in bone regeneration, a hydrogel scaffold with a biomimetic heterogeneous structure was designed to recreate the osteogenic activity of bone matrix and the angiogenic activity of periosteum. GelMA was reinforced with modified MBGN to generate a heterogeneous structured scaffold that mimicked bone matrix with superior structural stability and osteogenic potential. The GelMA was also incorporated with VEGF-loaded liposomes to guide angiogenesis in the inner and outer periosteum. After *in vitro* characterization of these two components individually, the combined heterogeneous structured scaffolds were further applied *in vivo* to treat critical-size bone defects in the rabbit radius (Figure 1).

## Results and Discussion

### Physicochemical Characterization

**FIGURE 2:**
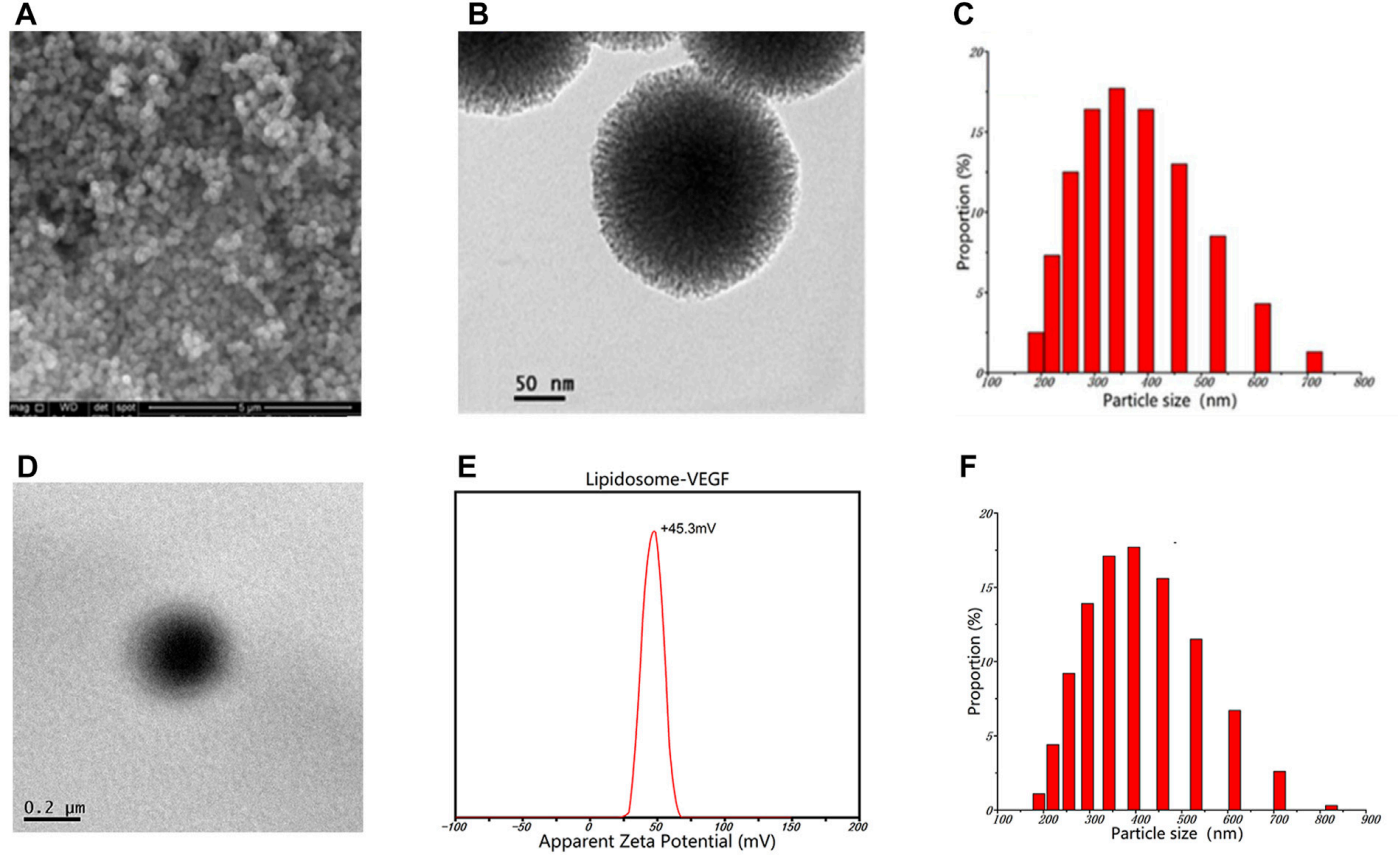
Characterization of G-MBGN and liposomes. **(A)** SEM observation of G-MBGN. Nanoparticles with relatively uniform sizes could be seen under a low magnification view. **(B)** Distinguishable mesopore inside nanoparticles under TEM. **(C)** DLS study on the particle size of G-MBGN and the particle size was around 340 nm. **(D)** TEM observation of the liposome. **(E)** DLS study on the zeta potential of the liposome. **(F)** DLS study on the particle size of the liposome.

As the essential elements incorporated in the outer and inner layer of the scaffold, mesoporous bioactive glass nanoparticles modified with GelMA (G-MBGN) and liposomes were synthesized for structural strengthening and drug loading respectively. Mesoporous bioactive glass nanoparticles modified with GelMA (G-MBGN) and liposomes were synthesized for structural strengthening and drug loading, respectively. The morphology of synthesized MBGN was observed *via* SEM. The synthesized nanoparticles showed relatively uniform size under low magnification (Figure 2A). Distinguishable mesopores were seen inside the nanoparticles using TEM (Figure 2B). The particle size of G-MBGN was analyzed using DLS. The results showed that the average diameter of the micro-sol particles was 340 nm, and indicated the uniformity of the particles (Figure 2C).

Liposome was prepared to load VEGF for controlled release. Before applying for drug loading, the morphology and encapsulating parameters of liposomes were investigated. The morphology of the liposome was observed using TEM. The results showed that liposomes loaded with VEGF exhibited a spherical morphology with a zeta potential of 45.3 mV and a diameter of around 400 nm (Figures 2D–F). The encapsulation performance of the liposomes was characterized by the encapsulation rate, which was found to be 62.4% ± 5.7%.

**FIGURE 3:**
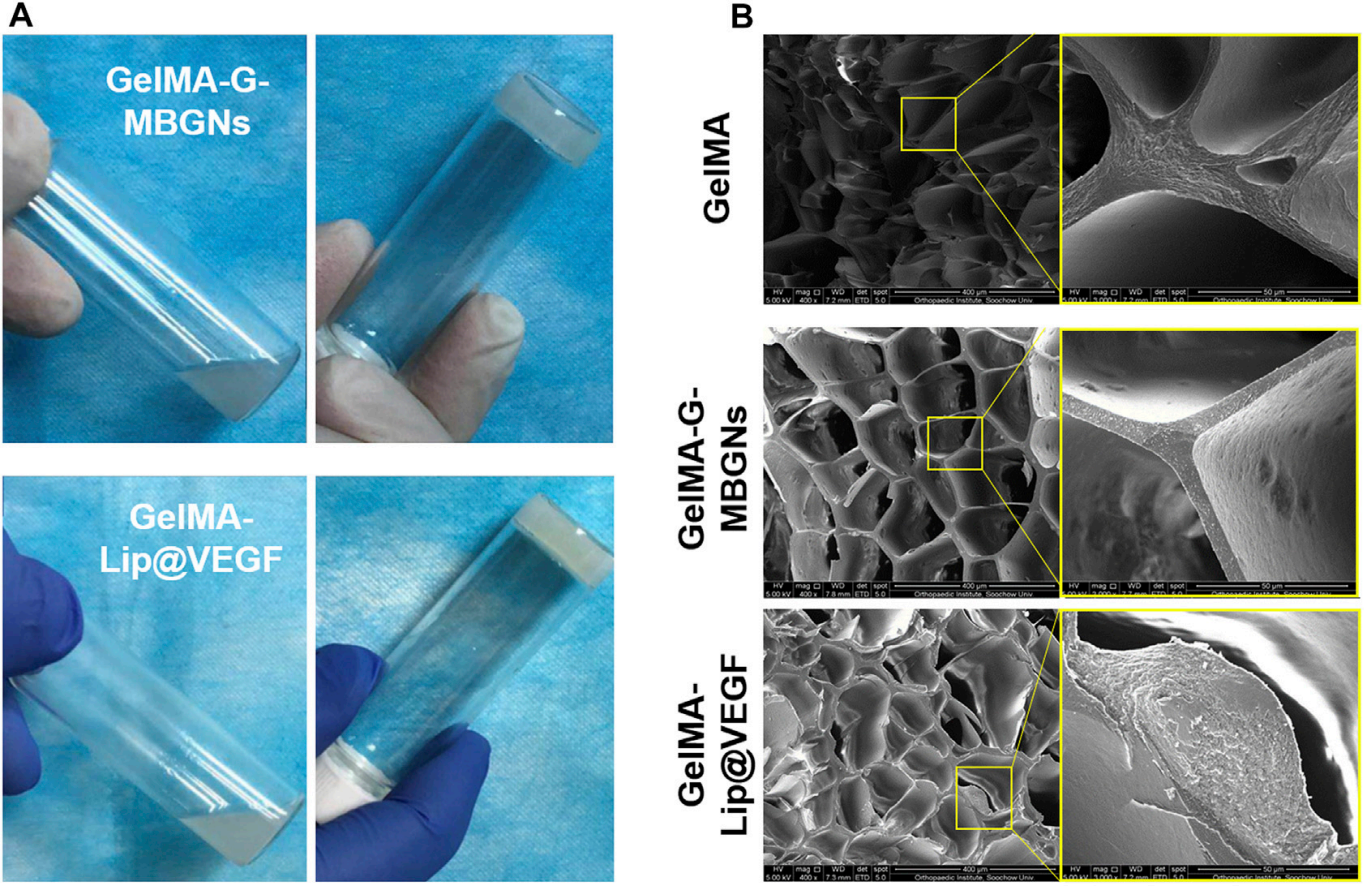
Characterization of the gelation process and microstructure. **(A)** Gelation process of GelMA-G-MBGN and GelMA-Lip were triggered by UV irradiation. **(B)** SEM observation of the microstructure.

After incorporating the G-MBGN and liposomes into the GelMA hydrogel, the composite hydrogel was also characterized by its morphological performance. The gelation process of GelMA-G-MBGN and GelMA-Lip was triggered after UV irradiation (Figure 3A). After gelation, both GelMA-M-MBGN and GelMA-Lip showed a porous structure with a smooth pore wall under SEM (Figure 3B). Compared with bare GelMA hydrogel, the addition of G-MBGN resulted in the same porous structure for different pore wall morphologies in the GelMA-G-MBGN hydrogel. White particles could be observed on the surface and section of the pore wall, which could be the incorporated MBGN. However, the particle’s appearance could also be observed on the pore wall of the GelMA-Lip porous structure, verifying the successful incorporation of liposome in GelMA-Lip hydrogel.

### Study on the Swelling Behavior and Drug Releasing Kinetics of GelMA-G-MBGN and GelMA-Lip@VEGF

The swelling behavior plays an important role in maintaining the shape and physical property of hydrogel materials implanted *in vivo*. Due to the high swelling ratio of the unmodified GelMA network, bare GelMA hydrogel could hardly represent a competent candidate as the outer layer of the heterogeneous scaffold. Therefore, based on the previous studies of our group on the mechanical strengthening of GelMA hydrogel, G-MBGN and liposome were respectively employed to improve the swelling performance of GelMA.

**FIGURE 4:**
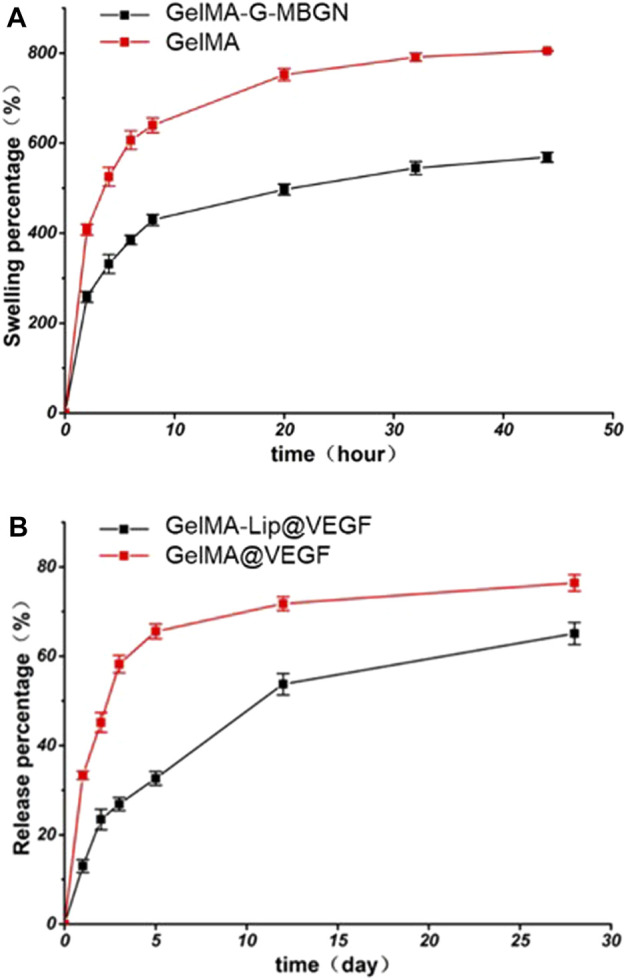
Characterization of the swelling behavior and drug release profile. **(A)** Swelling kinetics of hydrogels. **(B)** VEGF release profile from the hydrogel.

The effect of MBGN and liposome addition on the swelling behavior of GelMA hydrogel was studied by testing the swelling ratio on freeze-dried hydrogel samples after soaking them in PBS for a varying amount of time. The introduction of MBGN in the GelMA hydrogel has resulted in relatively lower swelling ratios in the hydrogel samples (Figure 4A). Specifically, both GelMA and GelMA-G-MBGN hydrogel samples achieved a stable swelling state after soaking for 48 h. Among them, the GelMA hydrogel exhibited a higher swelling ratio of 805.2% ± 10.4%, which could be attributed to the loose structure of bare GelMA. The GelMA-G-MBGN exhibited relatively lower swelling ratios, with GelMA-G-MBGN reaching 642.7% ± 9.7% after soaking for 48 h. The relatively lower swelling ratios could be attributed to the structural strengthening effect of G-MBGN on the GelMA hydrogel. Previous studies have verified the strengthening effect of MBGN on the structural integrity of hydrogels. G-MBGN could form covalent integration with the GelMA network, thus achieving a significant enhancement in structural stability as reflected by a lower swelling ratio compared with GelMA.

To endow the inner layer of the scaffold with effective angiogenic capacity, VEGF was loaded into GelMA-Lip hydrogel. However, burst release of VEGF from the loosened hydrogel matrix and the resulting limited half-life of VEGF at the focal area have significantly restricted the performance of VEGF-loaded hydrogel. Therefore, VEGF was loaded in liposomes embedded in GelMA hydrogel to achieve the controlled release kinetic of angiogenic factor. The release kinetics of VEGF from the liposome loaded in the GelMA-Lip@VEGF hydrogel were studied and compared with VEGF physically incorporated in the GelMA@VEGF hydrogel. Directly incorporated VEGF exhibited a burst release of 48.4% ± 4.1% within 5 days (Figure 4B). The GelMA@VEGF released 71.2% ± 5.9% of VEGF within 14 days of the release study, showing minor release in the later period. Conversely, VEGF loaded in GelMA-Lip@VEGF exhibited a significantly suppressed burst release activity, with 37.8% ± 4.1% of VEGF released in the first 5 days, and 58.5% ± 5.6% of VEGF released from GelMA-Lip@VEGF within 14 days, thus showing a controlled release profile compared with GelMA@VEGF. As one of the prevailing drug loading vehicles in tissue engineering, liposome has been employed for controlled release in various platforms ranging from hydrogel to electrospun scaffold. Under the current scenario, high water content and loosening of the structure of GelMA hydrogel could hardly achieve controlled release of physically incorporated drugs. Hence, liposome was introduced to act as the loading vehicle. Owing to the active interaction between liposome and GelMA molecular network evidenced previously, stable integration of liposome in GelMA-Lip hydrogel could be achieved as well as the lasting release of VEGF. The controlled release of the angiogenesis factor was essential for sustained vascularization in the focal area during bone regeneration (De la Riva et al., 2010; Knaack et al., 2014). GelMA-Lip@VEGF could exert stable angiogenesis activity and serve as the outer and inner periosteum.

### *In Vitro* Characterization of Biocompatibility

**FIGURE 5:**
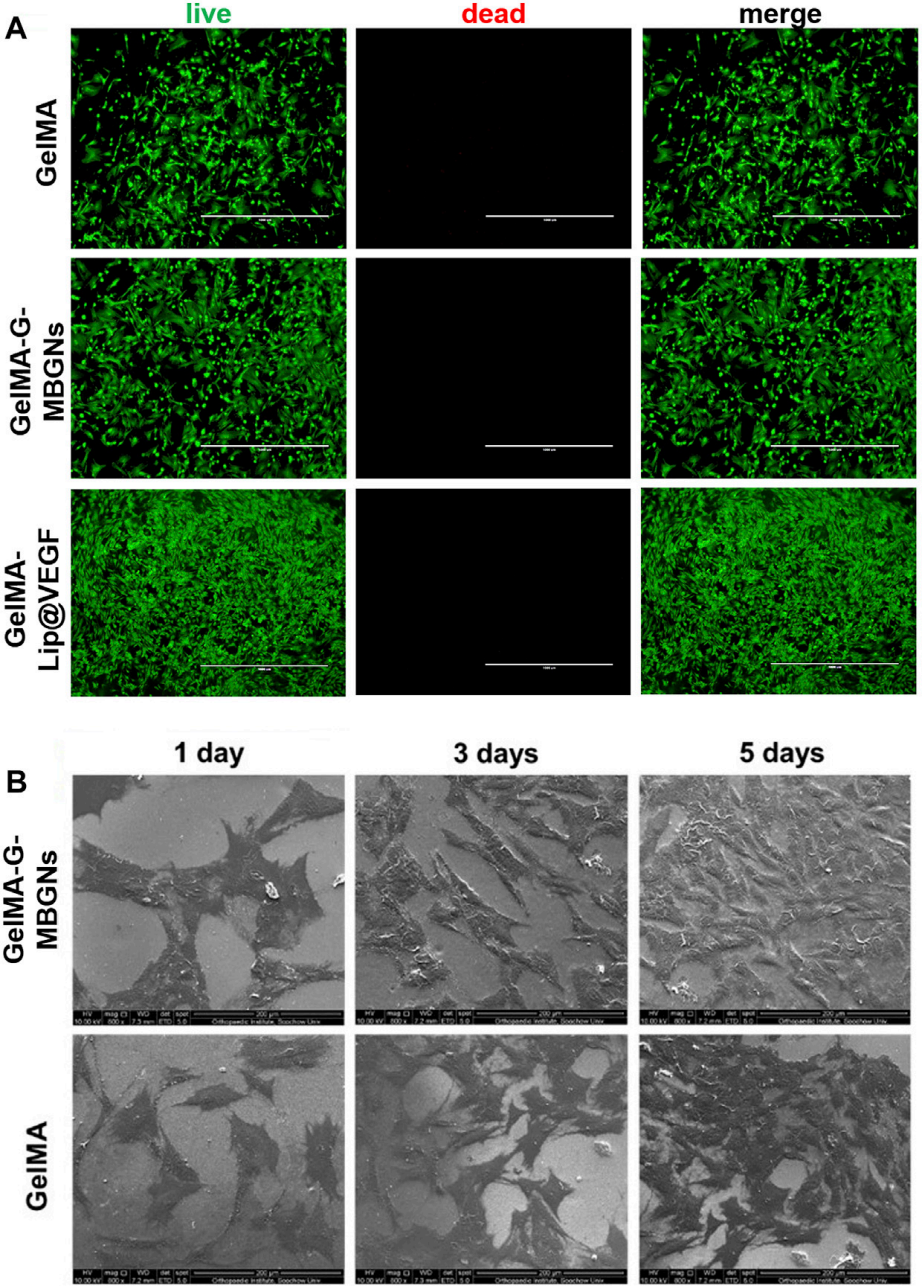
Characterization of biocompatibility. **(A)** Live/Dead staining of BMSCs seeded on hydrogels. **(B)** SEM observation of BMSCs on hydrogels.

The biocompatible performance of hydrogels is one of the decisive factors determining the initial effect after implantation. Although the biocompatibility of GelMA hydrogel has been thoroughly studied previously, the incorporation of G-MBGN and liposome could potentially jeopardize the original performance. Therefore, further investigation would be necessary for evaluation. The biocompatibility and bioactivity of GelMA-G-MBGN and GelMA-Lip hydrogel were assessed using *in vitro* experiments with BMSCs and HUVECs as model cells. The biocompatibility of different hydrogels was studied *via* seeding BMSCs on hydrogel samples. Cell viability and proliferation were investigated using Live/Dead staining and the CCK-8 test. The BMSCs seeded on GelMA, GelMA-G-MBGN, and GelMA-Lip exhibited adequate viability (Figure 5A). Adhesion and spread of BMSCs on hydrogels were also observed using SEM. The results showed that BMSCs exhibited a well-spread morphology on both GelMA-G-MBGN and GelMA hydrogels 1 day after seeding (Figure 5B). Additionally, significant proliferation of seeded BMSCs was observed as evidenced by a higher density of adhered cells compared with earlier time points (Figure 5B).

**FIGURE 6:**
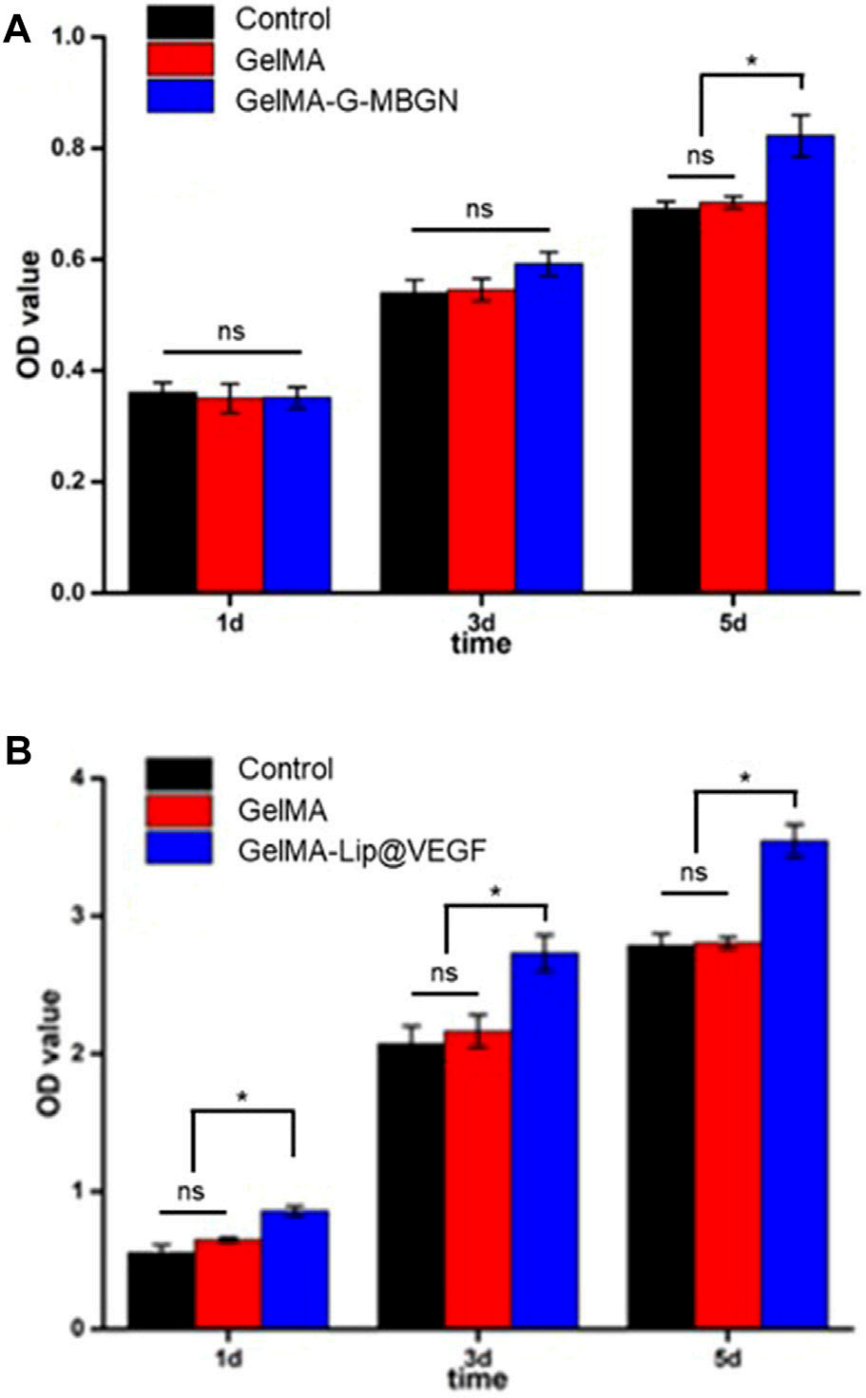
Characterization of the proliferation rate. **(A)** CCK-8 test for BMSCs seeded on the GelMA-G-MBGN hydrogel. **(B)** CCK-8 test for BMSCs seeded on the GelMA-Lip@VEGF hydrogel.

The result of the CCK-8 test showed the stable proliferation of BMSCs after seeding (Figure 6A). When compared with the GelMA and blank control group, the addition of G-MBGN resulted in a significant increase of cells on day 5 after seeding (Figure 6A). Moreover, the addition of liposomes loaded with VEGF promoted cell proliferation significantly (Figure 6B).

### Characterization of *In Vitro* Osteogenic and Angiogenic Potential From GelMA-G-MBGN and GelMA-Lip@VEGF

**FIGURE 7:**
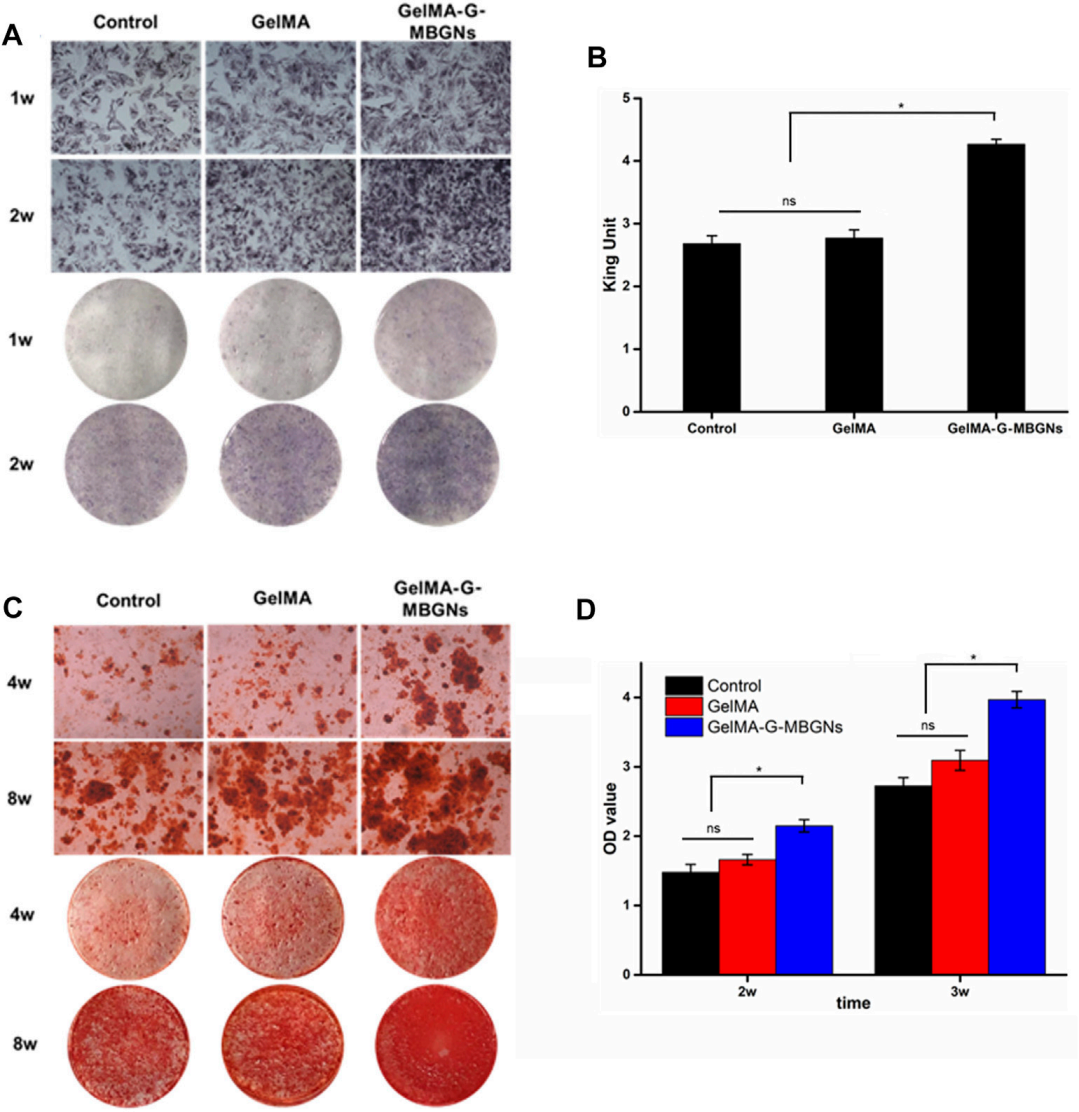
Characterization of the osteogenic activity. **(A)** ALP staining images for the microscopic and gross view. **(B)** ALP quantification test. **(C)** Alizarin red staining images for the microscopic and gross view. **(D)** ARS staining quantification.

Apart from serving as the intermediate layer of a structured scaffold to provide mechanical support, GelMA-G-MBGN was also expected to exert biological effects on progenitor cells and guide them towards osteogenic differentiation. The bioactive performance of the GelMA-G-MBGN and GelMA-Lip hydrogel was studied using *in vitro* experiments with BMSCs and HUVECs. BMSCs seeded on GelMA and GelMA-G-MBGN were cultured in an osteogenic medium for 2 weeks. ALP staining was conducted to investigate the early stage osteogenic activity of BMSCs. A higher staining intensity could be observed in BMSCs cultured on GelMA-G-MBGN compared with the GelMA and blank control group (Figure 7A). Next, an ALP quantification kit was employed to further investigate the ALP activity in the BMSCs on different hydrogels. A significantly higher quantified ALP value was observed on the GelMA-G-MBGN compared with the GelMA and blank control group 1 week after seeding (Figure 7B). The results indicated that GelMA-G-MBGN could induce a higher degree of ALP activity in BMSCs at an early stage of osteogenic induction. On the other hand, in addition to ALP staining and quantification, the formation of calcium nodules in BMSCs was also investigated as a late-stage osteogenic marker *via* Alizarin Red staining. In addition, calcium nodule formation in BMSCs was also observed *via* Alizarin Red staining. Denser calcium nodule staining could be observed on the GelMA-G-MBGN at 2 and 4 weeks after seeding compared with the GelMA and blank control group (Figure 7C). This indicates the superior performance of GelMA-G-MBGN in guiding BMSC calcium deposition. The corresponding quantification test revealed a higher OD value in the GelMA-G-MBGN group compared with the GelMA and the blank control group (Figure 7D).

**FIGURE 8:**
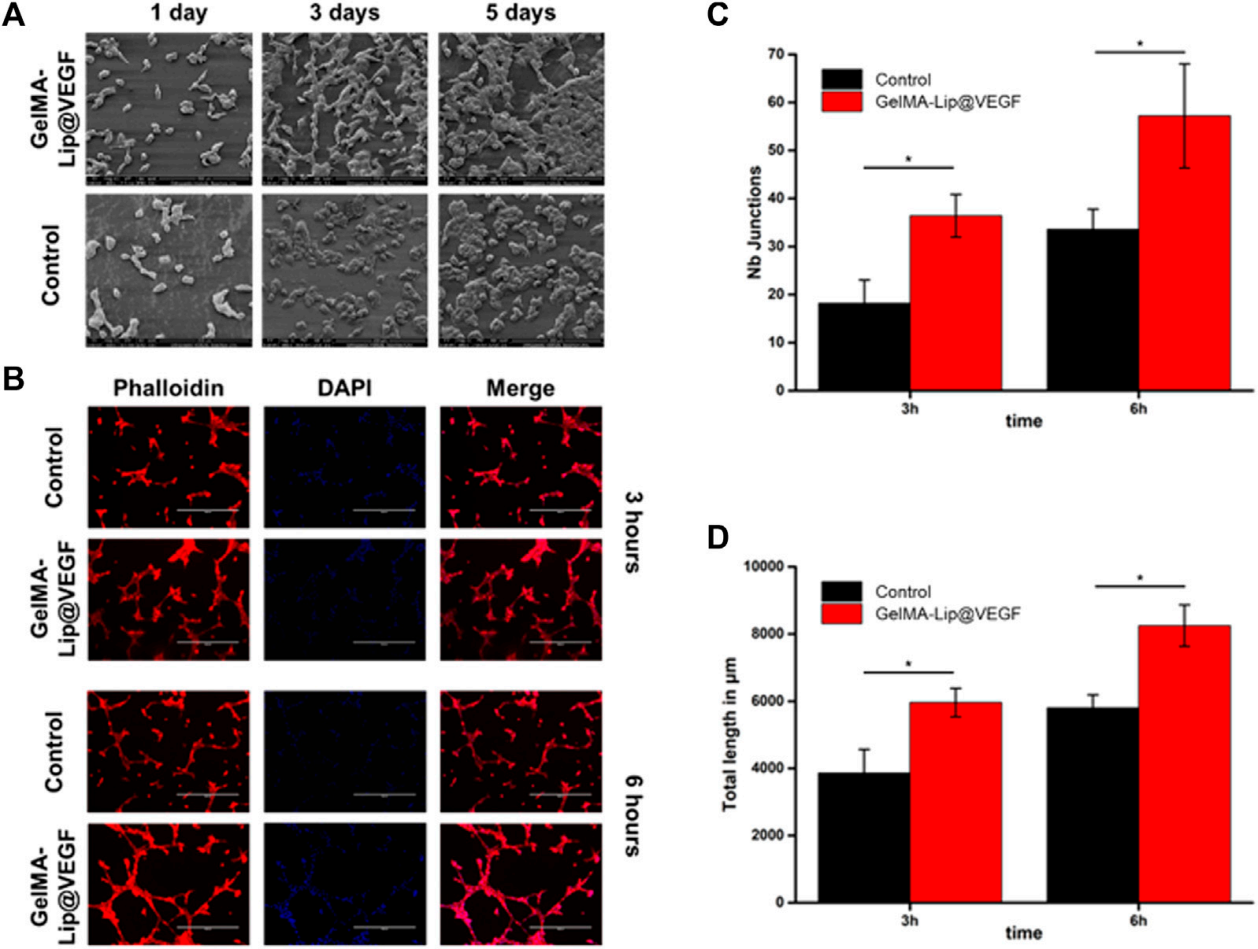
Characterization of the angiogenic activity. **(A)** SEM observation of HUVECs seeded on hydrogels. **(B)** Phalloidin and DAPI staining of HUVECs on hydrogels. **(C)** Quantification of the number of junctions. **(D)** Quantification of the tube length.

In addition to osteogenic activity, angiogenesis was also believed to be a pivotal activity in bone regeneration. In this study, by introducing GelMA-Lip@VEGF as the outer and inner periosteum of structured, enhanced vascular formation was expected to assist the superior bone repair capacity of the scaffold. The angiogenic activity of the GelMA-Lip hydrogel was characterized by seeding HUVECs on hydrogels. Based on the SEM observation, and phalloidin and DAPI staining, the HUVEC adhesion and spreading on GelMA and GelMA-Lip hydrogel was studied comprehensively. Well spread HUVECs could be observed on day 1 after seeding (Figure 8A). More HUVECs were observed at later time points, indicating the proliferation of HUVECs on GelMA and GelMA-G-MBGN hydrogels between these time points. Next, after staining with phalloidin and DAPI, HUVECs seeded on hydrogel were observed under a fluorescent microscope for the tube formation assay. Interaction between HUVECs could be seen on hydrogels, and vascular-like networks composed of numerous cell-cell interactions could be observed on the GelMA and GelMA-Lip hydrogels (Figure 8B). To quantitatively analyze the *in vitro* angiogenic activity of HUVECs under the influence of different hydrogels, the staining images were quantitatively analyzed using ImageJ software to obtain the angiogenic indexes including the number of tube junctions and tube length at different time points. More junction formations could be observed on the GelMA-Lip compared with the GelMA hydrogel (Figures 8C,D). Additionally, a significantly longer tube length could be found on the GelMA-Lip, indicating its superior angiogenic potential *in vitro*.

### Construction of a Biomimetic Heterogeneous Structured Scaffold

**FIGURE 9:**
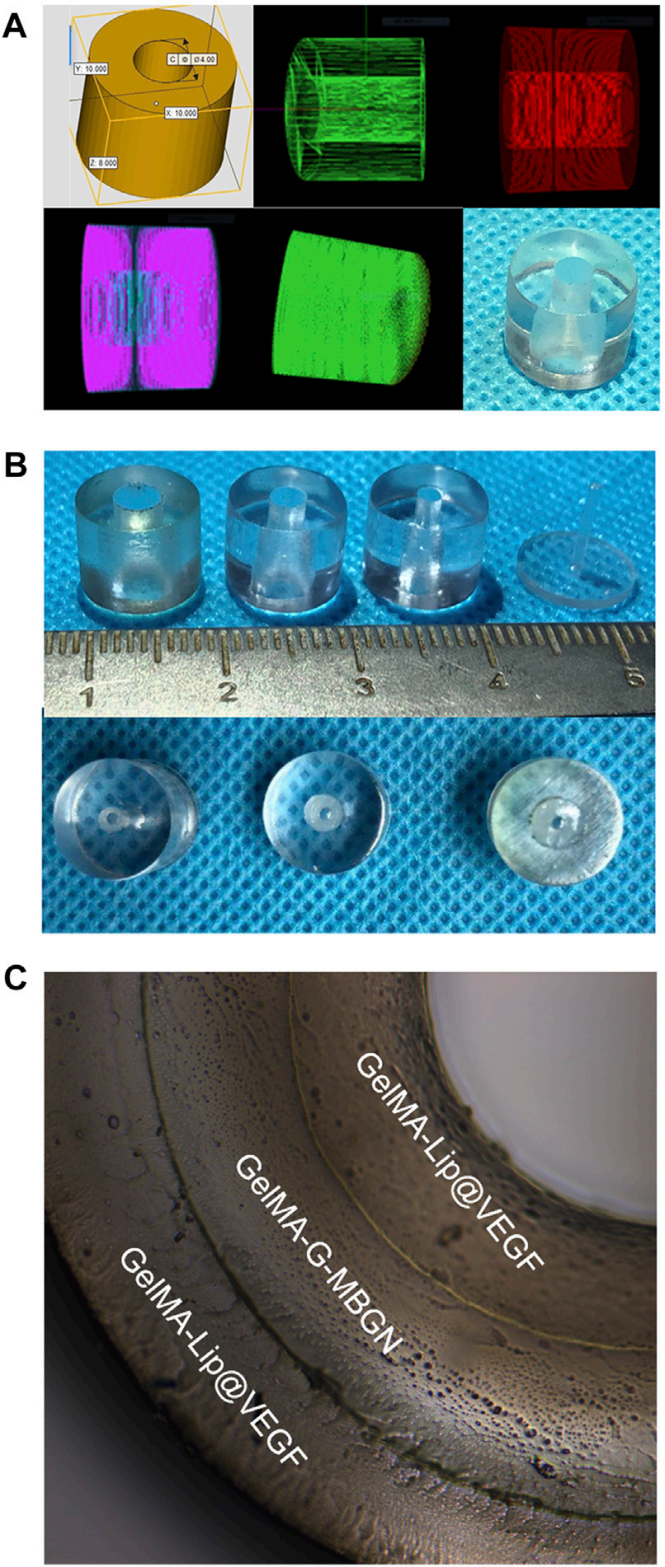
Preparation of the 3D print mold and structured scaffold. **(A)** The digitally programmed shape of the 3D print mold. **(B)** Gross observation of the molds. **(C)** The inner, intermediate, and outer layers of the structured scaffold.

After thorough characterization of the respective performance of GelMA-G-MBGN and GelMA-Lip hydrogels, the biomimetic structured scaffold with heterogeneous architecture was built with the help of a 3D-print mold (Figures 9A,B). Molds with different inner diameters were employed to construct the structured scaffold’s inner, intermediate, and outer layers (Figure 9C). The constructed structured scaffold was observed under optical microscopy. The structured scaffold had the shape of the bone piece defect of the fractured radius. The cross-section view of the scaffold under optical microscopy also verifies the biomimetic sandwich-like structure consisting of an inner and outer GelMA-Lip layer coupled with an intermediate GelMA-G-MBGN layer. The structured scaffold would be further subjected to *in vivo* study to investigate its performance in promoting repair of bone defects.

### *In Vivo* Characterization of the Structured Scaffold *via* the Rabbit Radius Critical-Size Bone Defect Model

**FIGURE 10:**
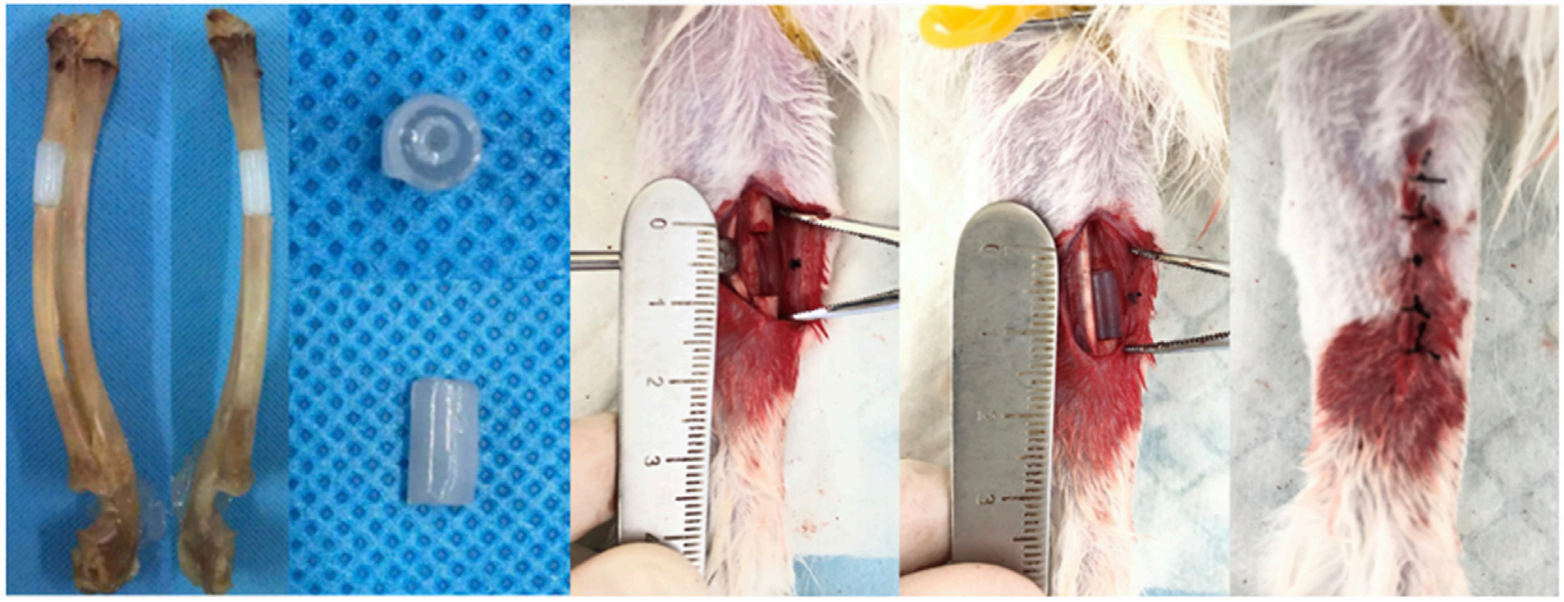
Preparation of the rabbit radius critical-size bone defect model and scaffold implantation.

In order to study the *in vivo* performance of biomimetic heterogeneous scaffold in promoting bone regeneration, a rabbit radius critical-size bone defect model was prepared to investigate the *in vivo* performance of a structured scaffold (Figure 10). The structured scaffold and bulk hydrogel were implanted in the defect area to guide bone regeneration.

**FIGURE 11:**
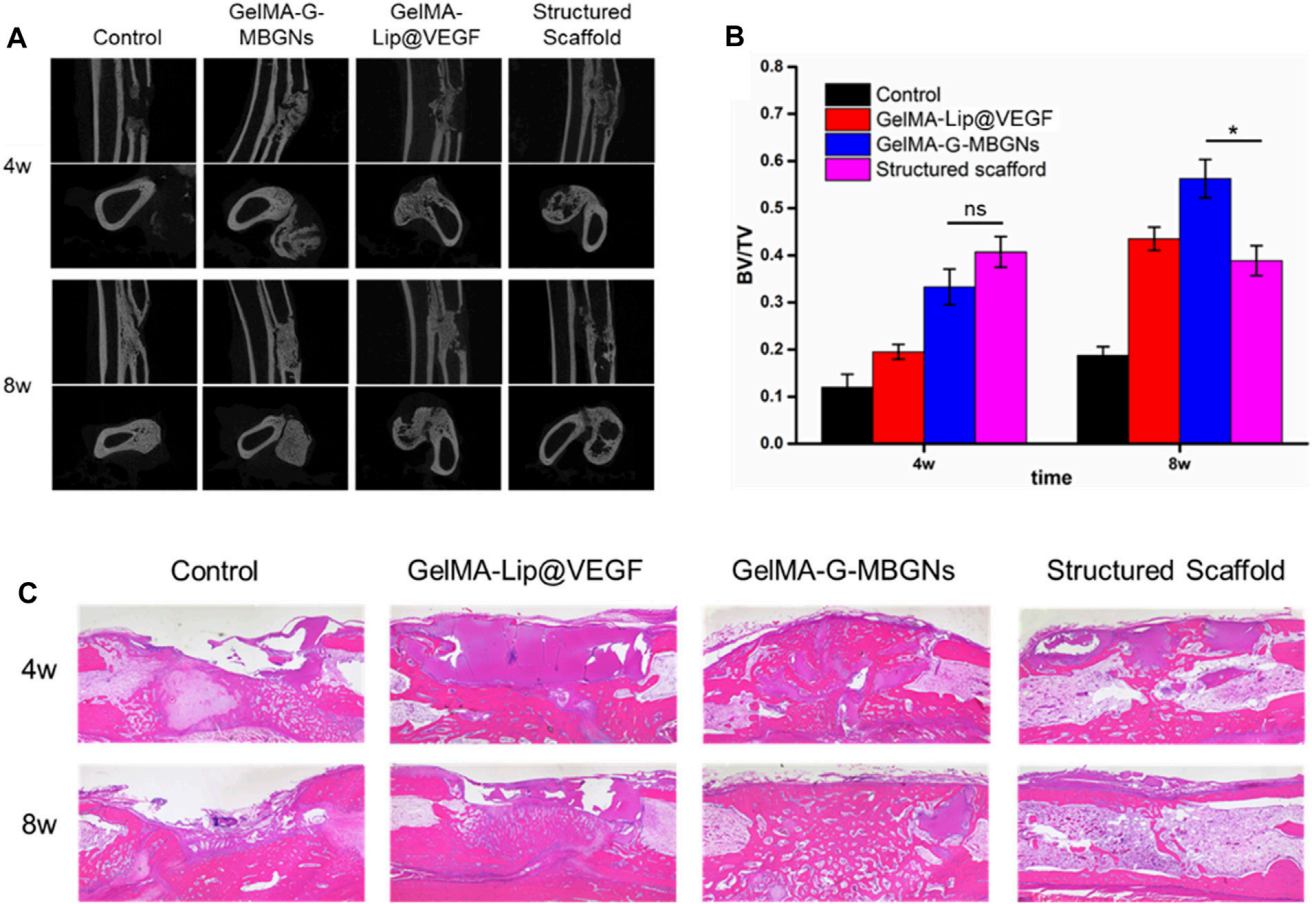
Radiological and pathological assessment of the animal model. **(A)** Micro-CT observation of the bone defect. **(B)** Quantified BV/TV analysis. **(C)** H&E staining of the samples.

At 4 and 8 weeks after surgery, the rabbits were euthanized to harvest radius-ulna samples subjected to micro-CT scanning. The coronal and axial sections of the radius and ulna were reconstructed to show the details of the bone defect (Figure 11A). Varying regeneration activities could be found in all groups. Four weeks after surgery, the blank control group induced negligible regenerated bone because of the critical size of the bone defect. Limited regeneration could be observed at the defect area 8 weeks after surgery and bone marrow cavity closure in the blank control group. Conversely, groups receiving the hydrogel scaffold achieved different healing outcomes. The bone defect treated with the GelMA-Lip bulk hydrogel produced limited new bone, leaving most defect areas filled with undegraded hydrogel 4 weeks after surgery. At 8 weeks after surgery, in spite of the bony connection achieved at the ulnar side of the radius, the bone defect remained at the radial side of the radius which was occupied by the undegraded hydrogel. For the group receiving the GelMA-G-MBGN bulk hydrogel scaffold, active osteogenesis activity was observed at both 4 and 8 weeks after surgery. However, despite abundant bone regeneration in the defect area, bone marrow cavities from two sides of the fracture did not form a connection. In comparison, a structured scaffold composed of GelMA-G-MBGN and GelMA-Lip induced bone regeneration on the radial and ulnar sides of the defect area. The bone marrow cavity could be reconnected at 8 weeks after surgery with the scaffold wholly degraded in the defect area, thus achieving the natural anatomic structure of the radius. A corresponding quantification study also revealed a similar trend (Figure 11B). Although the structured scaffold did not result in a significantly higher BV/TV ratio compared with GelMA-G-MBGN bulk materials, the restoration of the natural anatomic structure still indicated the superior treatment efficiency of the structured scaffold.

The pathological study using H&E staining also revealed similar results to the micro-CT. Although active osteogenesis could be found in GelMA-G-MBGN and GelMA-Lip hydrogels, recanalization of the medullary cavity was not achieved in these two groups (Figure 11C). In comparison, owing to the biomimetic heterogeneous architecture of structured scaffolds, the group receiving structured scaffolds exhibited superior regeneration outcomes (Figure 11C). The reunion of radius on both ulnar and radial sides as well as the recanalization of radius was achieved at 8 weeks after surgery (Figure 11C).

As one of the most difficult tasks in tissue engineering, repair of critical-sized bone defects has remained a tough challenge for most biomaterials. However, due to the increasing demand for healing quality, even more requests have been put forward in treating such conditions. For instance, the healing of bone defect and the reconstruction of normal anatomic structure has been highlighted recently. Both successful healing and recanalization of long bone should be achieved. Traditional bulk materials with homogeneous structures could hardly reproduce the heterogeneous structure of natural bone. In this study, relying on the heterogeneous structured scaffold, the healing and recanalization of critical-sized bone defect were achieved and such design could represent a novel strategy for treating bone defects in the future.

## Conclusion

This study employed GelMA-G-MBGN to mimic the bone matrix while GelMA-Lip loaded with VEGF was introduced to act as the inner and outer periosteum. A biomimetic, heterogeneous, structured scaffold reproducing the natural bone structure was constructed for the regeneration of the critical-size bone defect. Physiological characterization and *in vitro* experiments demonstrated that GelMA-G-MBGN had stable structural integrity and the potential for promoting osteogenesis. GelMA-Lip loaded with VEGF was also found to exhibit the controlled release of loaded VEGF and exert effective angiogenesis activity *in vitro*. By combining GelMA-G-MBGN and GelMA-Lip@VEGF, the structured scaffold was successfully built with the help of a 3D printing mold. After fitting this scaffold into the critical-size radius bone defect, regeneration of bone defects with recanalization of the medullary cavity could be achieved in a rabbit model, thus verifying the superior performance of the biomimetic heterogeneous structured scaffold.

## Materials and Methods

### Synthesis of Gelatin Methacryloyl

The synthesis of gelatin methacryloyl was conducted according to a previously reported procedure (Hutson et al., 2011). In brief, gelatin (20 g) was dissolved in PBS (200 ml) in a 60°C water bath. Methacrylic anhydrides (16 ml) were then added to the gelatin solution using a syringe pump (speed: 0.25 ml/min). After the injection, the reaction was allowed to continue for 2 h (under stirring conditions). Next, PBS (800 ml), preheated to 50°C, was added to the reaction, followed by a further reaction for 15 min. For 1 week, the resulting mixture was dialyzed against deionized water in a dialysis tube (cut-off MW: 8,000–14,000). After dialysis, the product was filtered to remove precipitates and freeze-dried for future use.

### Synthesis of Mesoporous Bioactive Glass Nanoparticles

The mesoporous bioactive glass nanoparticles were synthesized according to previous reports (Xin et al., 2017; Xin et al., 2020). In brief, the reaction was conducted in Tris-HCl buffer solution (pH 8.0) with cetyltrimethylammonium bromide (CTAB) as a templating agent. Then, tetraethyl orthosilicate (TEOS; 16 ml), triethyl phosphate (TEP; 1.22 ml), and calcium nitrate tetrahydrate (CN; 3.39 g) were added sequentially into the buffer solution (and the reaction was completed in a 60°C oil bath). The mixture was allowed to react for 24 h (at 60°C), and the produced nanoparticle was recovered using a centrifuge (at 12000xg). This nanoparticle was further washed (three times) with ethanol and deionized water. The final product containing SiO_2_ (80 mol%), CaO (16 mol%), and P_2_O_5_ (4 mol%) was obtained *via* nanoparticle sintering at 650°C for 3 h.

### Synthesis of GelMA-Conjugated MBGN (G-MBGN)

Before synthesizing G-MBGN (Xin et al., 2020), the MBGN was functionalized with amine groups. In brief, MBGN (0.4 g) was dispersed in hexane (100 ml) and aminopropyltriethoxysilane (APTES; 5 ml) for 24 h at 60°C. The amine-functionalized MBGN (A-MBGN) was obtained after washing and drying at 60°C. The GelMA-G-MBGN was synthesized by dispersing MBGN (0.3 g) into deionized water (20 ml) containing GelMA (0.3 g) and reacting with 1-Ethyl-(3-dimethylaminopropyl)carbodiimide hydrochloride (EDC; 0.4 g) and N-Hydroxysuccinimide (NHS; 0.2 g) for 24 h. The GelMA-G-MBGN was obtained after washing and drying the product.

### Synthesis of the VEGF-Loaded Liposome

The drug-loaded liposome was prepared *via* phacoemulsification (Cheng et al., 2018). In brief, soy lecithin (160 mg), cholesterol (40 mg), and octadecylamine (5 mg) were added into ether (6 ml). After oscillation to obtain the solution, VEGF (500 μg) was added into deionized water (2 ml) and mixed with the ether-based solution. The resulting mixture was emulsified *via* ultrasonication and then subjected to rotary evaporation under an ice bath. After the evaporation of ether, the VEGF-loaded liposome was obtained through freeze-drying.

### Preparation of Gelatin Methacryloyl, GelMA-G-MBGN, and GelMA/Lip Hydrogel

The GelMA hydrogel was prepared by dissolving GelMA (10% w/v) and photoinitiator Irgacure 2959 (1% w/v) in PBS using a 60°C water bath. The solution was cured into the GelMA hydrogel *via* ultraviolet irradiation (10 cm W/cm^2^) for 3 min.

The GelMA-G-MBGN hydrogel was prepared using the previously prepared GelMA and G-MBGN. Briefly, GelMA (10% w/v), G-MBGN (3% w/v), and the photoinitiator Irgacure 2959 (1% w/v) were added to the PBS and sonicated at 60°C to dissolve the GelMA and photoinitiator and disperse the G-MBGN. After achieving the stable suspension of G-MBGN in GelMA solution, the mixture was subjected to ultraviolet irradiation (10 cm W/cm^2^) to obtain the GelMA-G-MBGN hydrogel.

The GelMA/Lip hydrogel was prepared by dissolving GelMA (10% w/v) and photoinitiator Irgacure 2959 (1% w/v) in PBS at 60°C. The VEGF-loaded liposome was added to the solution and cooled down to room temperature. After full dissolution, the solution was subjected to ultraviolet irradiation (10 cm W/cm^2^), and the GelMA/Lip hydrogel was obtained.

### Physical Characterization of Nanomaterials and Hydrogels

Scanning electron microscopy (SEM, S-4800, Hitachi, Japan) was employed to observe the microstructure of MBGN and GelMA-based hydrogels. The freeze-dried samples were fixed on the sample stage using conductive tape. The SEM observation was conducted at a voltage of 5 kV after gold coating for 60 s using sputter coating equipment (SC7620, Quorum Technologies, United Kingdom). A transmission electron microscope (TEM) was employed to observe the detailed structure of MBGN and liposome.

A swelling test was applied to study the swelling behavior of hydrogels. In brief, freeze-dried GelMA and GelMA-G-MBGN hydrogel samples were weighed and immersed in PBS and then fixed on a shaker at 37°C. The swelled weights were measured at a specific time to calculate the swelling ratios.

The encapsulation efficiency of liposomes and the release kinetics of VEGF from liposome and hydrogel were studied using an Enzyme-linked immunosorbent assay (ELISA) kit. In brief, the encapsulation rate was studied (using an ELISA kit) by measuring the unencapsulated particles in the supernatant of the liposome-VEGF solution after centrifugation. The release kinetics of VEGF from liposome and hydrogel was studied *via* immersion of the samples in PBS solution. This was performed in a 37°C shaker with a rotating speed of 100 rpm. The released VEGF content was determined by measuring the PBS samples at different time points using an ELISA kit.

### *In Vitro* Characterization of the Hydrogel

The biocompatibility of GelMA, GelMA-G-MBGN, and GelMA-Lip hydrogel was studied by *in vitro* characterization using bone marrow mesenchymal stem cells (BMSCs) and human umbilical vein endothelial cells (HUVEC). The characterization was conducted by seeding BMSCs onto different hydrogels and testing the spreading, viability, and proliferation of cells at different time points using SEM observation, Live/Dead staining kit, and CCK-8 kit. Specifically, SEM observation of the adhesion and spreading conditions was conducted after seeding cells onto hydrogels and culturing for 5 days. The hydrogel-cell samples were fixed using PFA (4%) and further dehydrated *via* a gradient ethanol solution. The sample was subjected to SEM observation with a voltage of 5 kV after gold coating for 75 s. The viability of cells on the hydrogel was studied using a Live/Dead staining kit (Invitrogen, United States) 5 days after seeding. A CCK-8 kit (Beyotime, Shanghai) was employed to study the BMSC proliferation rate on different hydrogels after culturing for 1, 3, and 5 days.

The *in vitro* bioactivity performance of GelMA-G-MBGN and GelMA-Lip hydrogel on osteogenesis and angiogenesis was investigated. The osteogenic activity of BMSCs cultured on GelMA and GelMA-G-MBGN at an early stage was characterized after seeding BMSCs and culturing in osteogenic media for 7 and 14 days using the ALP staining kit (Beyotime, Shanghai) and ALP quantification kit (Jiancheng, Nanjing). The osteogenic activity of BMSCs at later time points was measured using an Alizarin Red staining kit (Beyotime, Shanghai) after seeding BMSCs on hydrogels and culturing in osteogenic media for 14 and 21 days. A corresponding quantification study was also carried out using perchloric acid to dissolve calcium nodules and further tested for OD value (wavelength: 562 nm).

The angiogenesis of HUVECs on GelMA and GelMA-Lip hydrogels was evaluated by phalloidin and DAPI staining at 3 and 6 h after seeding. The quantification of parameters involved in tube formation based on phalloidin staining of HUVECs was conducted using ImageJ software (United States) to evaluate the angiogenic activity on different hydrogels.

### Construction of a Biomimetic Structured Scaffold

A scaffold with a biomimetic heterogeneous structure was constructed with the assistance of three-dimensional (3D)-printed moldings. In brief, the rabbit radius and ulna complex were harvested from male New Zealand rabbits weighing 2.5 kg. A critical-sized radius bone defect with a length of 1.5 cm was prepared using a swing saw on the middle shaft of the radius. The structural parameters of the obtained bone sample were measured for customized 3D printing by the NovaPrint company. A 3D-print mold was prepared for the casting of the biomimetic structured scaffold. The previously prepared GelMA-Lip hydrogel was employed to cast the inner and outer layers of the structured scaffold. Finally, the GelMA-G-MBGN was used to cast the intermediate layer of the scaffold.

### Animal Surgery

To characterize the *in vivo* performance, a male New Zealand white rabbit was used to prepare a critical-size bone defect model. All animal experiments conducted in this study, including surgical procedure, perisurgical handling, and postsurgical harvesting, were carried out following the guidelines approved by the Ethics Committee at the First Affiliated Hospital of Soochow University.

The rabbit radius critical-size bone defect model was created according to a previously described procedure (Meinig et al., 1996). In brief, general anesthesia was carried out on rabbits using an intramuscular injection of pentobarbital sodium (60 mg/kg). After skin preparation and disinfection on the forearm, a longitudinal incision was created to expose the radius shaft through blunt separation. A bone defect with a length of 1.0 cm was created using a swing saw. After proper hemostasis, the hydrogel scaffolds were placed in the defect sites, and then the wounds were closed and sutured layer-by-layer. Post-surgery, 8 × 10^5^ U penicillin per day was applied to the rabbits to prevent infection.

### Micro-CT Study

The rabbits were euthanized *via* air embolism at 4 and 8 weeks after surgery. The radius-ulna complex was harvested for characterization using micro-CT scanning. The samples were scanned at a resolution of 9 μm with an Al filter (1 mm), and the parameters applied in the examination were 65 kV and 385 mA. The coronal, sagittal, and axial views of the radius-ulna complex were reconstructed to observe bone regeneration in defective areas. The morphological details in the bone defect area was further studied using CTan software (Bruker). The bone volume (BV)/total volume (TV) parameter was calculated in the cylindrical region of interest (ROI) covering a defect area (diameter: 0.5 cm and length: 1 cm).

### Pathological Assessment

The pathological details of the defect area were studied using H&E staining. In brief, the radius-ulna sample was decalcified by soaking in an EDTA decalcification solution (Yuanye, Shanghai) for 4 weeks. After decalcification, the sample was further dehydrated and embedded for slicing. Slices (thickness: 8 μm) were prepared for staining using an H&E staining kit (Beyotime, Shanghai). The stained slides were scanned and observed using CaseViewer software.

### Statistical Methods

All data in this study were presented in the form of mean ± standard deviation. Statistical analysis was carried out using ImageJ and GraphPad Prism 7. A difference with a *p*-value less than 0.05 was considered statistically significant.

## Data Availability

The original contributions presented in the study are included in the article/Supplementary Material; further inquiries can be directed to the corresponding authors.
